# 

**Published:** 1888-07

**Authors:** 


### EDITORIAL.

### Ohio State Dental Society.

The fourth annual meeting of the Ohio State Dental Society, (reorganized) will be held in Cincinnati, October 16, 17, 18, 1888. Every effort will be made to make this the best meeting ever held in the State. JERE E. ROBINSON, President.
Hillsboro, Ohio. J. R. CALLAHAN, Secretary.

### American and Southern Dental Associations.

The joint meeting of these two bodies will be held at Louisville, Ky., beginning Tuesday, August 28th, 1888. This is the twenty-eighth annual meeting of the American Dental Association, and the twentieth annual meeting of the Southern Dental

Association. A most excellent programme has been prepared by the joint committees of the two associations and published. This programme arranges for a full occupancy of the time for five days; a morning session from nine to one; afternoon at three o'clock to half past five will be devoted to separate meetings of the sections for routine business and the meetings of various committees. There will also be a joint meeting each evening at 7.30 o'clock. The probabilities, and indeed the indications are that this will be one of the largest convocations of the Dental profession of the United States ever held. Doubtless the various committees will vie with each other in the preparation and presentation of their work. Clinics will be held in the afternoon of each day and on Saturday at 8.30 A. M. to 1.30 P. M. This will be an epoch in the history of the Dental profession of this country; a meeting in which every member of the profession who has any pride whatever in his profession should take a deep interest and so far as possible be present; and it is to be hoped that all the members will come prepared to add something in one way or another to the interest and profit of the occasion. Dr. Frank Abbott, of the American, and Dr. B. H. Catching, of the Southern, will preside alternately over the meetings. We doubt not the profession will receive an impetus from the meeting of these bodies that will aid greatly for good. Arrangements have been made for a one and a third fare for the round trip on the usual certificate plan. Every one taking advantage of this rate should procure a certificate at his ticket office before starting from home. That being signed by the Secretary of the Association enables the holder to return at one third fare. Quite reduced rates have been made by the various hotels of Louisville for those attending the meeting.

### Mad River Valley Dental Association.

The annual meeting of this society, which should have taken place at Dayton, Ohio, about the 18th of May, was postponed for reasons which seemed quite sufficient to the officers of this society. This postponement, though unavoidable, all the members no doubt regretted. Whether a meeting will be held this fall, or deferred till the period of the next annual meeting, we believe has not yet been decided. Postponements of this kind are always adverse to the interests and well-being of any such body, and should be avoided whenever possible. We hope when it is held that the officers will anticipate that meeting sufficiently to issue a good programme. Gentlemen, do not let the Mad River flag.

### Rhinological Association.

The sixth annual meeting of the American Rhinologial Association will be held at Cincinnati, Ohio, September 12th, 13th, and 14th.

It is to be hoped that there will be a large attendance of those interested. DR. JOHN NORTH, Secretary, Keokuk, Iowa.

### Hymenial.

Married on June 27th, 1888, at Akron, Ohio, Dr. Will. H. Witslar, of Youngstown, Ohio, to Miss Nellee M. Chisnell of the former place.

Dr. W. may consider that he has the congratulations, through this medium, of all his friends, and especially of those who were his associates in the University of Michigan; the Boys all wish him a happy life in his new estate; and that in it, he will make an eminent success, as he does in all things that he undertakes. May connubial bliss, with all that means, be his to the full.

### THE DENTAL REGISTER,

**A MONTHLY MAGAZINE DEVOTED TO THE INTERESTS OF THE**

### DENTAL PROFESSION.

*Terms Reduced to* $2.00 *Per Year. Single Copies,* 25 *Cents.*

### EDITED BY PROF. J. TAFT, D.D.S.

—AND—

### Published by SAM'L A. CROCKER & CO., Ohio Dental and Surgical Depot.

The volume commences in January and closes in December.

The REGISTER being issued monthly is always fresh, therefore Indispensable to the practitioner who desires to keep posted up with the new inventions and improvements which are daily developed. Neither expense nor effort will be spared to give value and variety to its contents. Articles will be illustrated with well executed engravings whenever they will add to the interest or assist to explanation.

Its extensive circulation and frequency of issue make it one of the BEST DENTAL ADVERTISING MEDIUMS in the United States.

All subscriptions must begin with January or July.

Local or special notices, five lines or less, will be inserted for $3 each insertion; over five lines, 50 cts. per line.

All advertising bills payable in advance, or at furthest, after the issue of the first number containing the advertisement. All transient advertisements to be paid for in advance.

☞CONTRIBUTIONS SOLICITED.

Exclusively Editorial Communications should be addressed to the Editor.

The latest number of the REGISTER may be ordered with or without other goods at any time, and all letters should be addressed to

**SAM'L A. CROCKER & CO., Ohio Dental and Surgical Depot, Cincinnati, O.**

